# Compressor Power and Efficiency Optimization: A Finite-Time Thermodynamics Approach

**DOI:** 10.3390/e27080842

**Published:** 2025-08-08

**Authors:** François Lanzetta

**Affiliations:** CNRS, Institut FEMTO-ST, Université Marie et Louis Pasteur, F-90000 Belfort, France; francois.lanzetta@univ-fcomte.fr; Tel.: +33-384-578-224

**Keywords:** finite time thermodynamics, compressor, irreversibility, optimization, efficiency

## Abstract

This paper presents a theoretical optimization of an endoreversible compressor under steady-state conditions. A parametric study using finite-time thermodynamic principles highlights the effect of external irreversibilities on compressor performance. A compressor efficiency metric is established based on heat pump theory’s analogous performance coefficient concept. The external irreversibilities are characterized as functions of the conductance coefficients between the compressor and the low- and high-pressure reservoirs. In particular, the influence of suction and discharge tube diameters and gas pressures is investigated to determine the optimum compressor operating performance for a given gas mass flow rate. The results highlight the importance of selecting optimal suction and discharge tube diameters to improve compressor power efficiency and minimize energy consumption during gas compression.

## 1. Introduction

The optimization of thermal machines has been the subject of numerous engineering studies and has accompanied their industrial development since the late XIXth century. In the 1950s, Chambadal and Novikov optimized thermal machine cycles by considering irreversibilities at the heat exchangers [1,2], leading to an efficiency depending only on the temperatures of the hot source and cold sink. A few years later, Curzon and Alborn laid the groundwork for finite-time thermodynamics [3]. Their article obtained the same results as those of Chambadal and Novikov. Their general approach was to treat time as a finite quantity during each cycle transformation and to incorporate internal and external irreversibilities in the fluid and heat exchangers. Since then, FTT has gone from strength to strength [4,5,6,7,8,9,10]. The introduction of finite-time thermodynamics has made it possible to model and optimize thermal machine cycles, starting with Carnot engines [11,12]. Optimal performance metrics, such as efficiencies and power outputs, are determined as a function of heat source and heat sink temperatures. This methodology has been extended to numerous thermal machines, such as Carnot heat machines [13,14,15,16,17,18,19,20], internal combustion engines [21], external heat input machines [11,12,22,23,24,25], turbines [21,26,27], co-generation systems [27,28,29], fuel cells [25,28], refrigeration machines [30,31,32,33], heat pumps [34,35,36], and combined cycles [29,32,37]. The notion of finite time was then subsumed under a more general terminology: finite dimension. This dimension can also be the speed of a piston [38,39,40,41] or the surface area of an exchanger [32]. Generally speaking, finite-time thermodynamics can be applied to any energy conversion process that generates work from hydraulic, pneumatic, chemical, thermal, or electrical potential differences, and vice versa. Examples include thermoelectrical systems [42,43] and pneumatic cylinders [10,44,45,46,47]. The study of fluid flow in microchannels is relevant to both industry and research, with applications spanning biology, chemistry, microelectronics, space, and micromechanics [48,49]. These microsystems involve creating geometric objects with dimensions as small as a few microns. Under these challenging flow conditions, actuators like valves, pumps, and distributors have operational characteristics that depend heavily on the dimensions and physical properties of the fluids, whether liquid or gas.

In this article, we explore the theoretical study of the optimal performance of a compressor used in high-pressure micro gas flow scenarios. Such issues arise particularly in the cooling of electronic components using thermal machines like pulsed gas tubes, Stirling refrigerators, or Joule Thomson refrigerators [50,51]. We explore the theoretical analysis, drawing a parallel with the finite-time thermodynamics of a heat pump to determine the optimal operating conditions of a compressor functioning under stringent geometric constraints, necessitating suction and discharge flows through tubes with internal diameters ranging from 70 to 200 µm.

## 2. Mathematical Model

**Figure 1:**
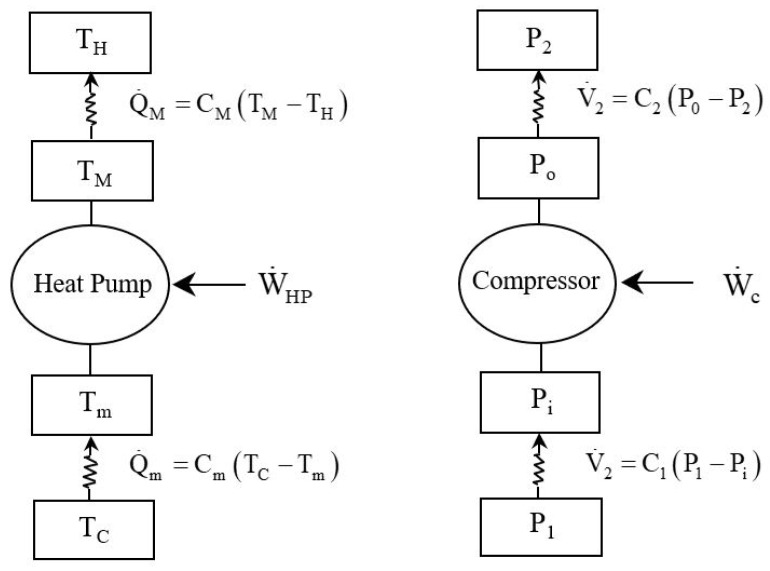
Analogical representation of endoreversible heat pump and an analog endoreversible compressor.

Bejan [10] presented a study on the maximum power generated by fluid flow, using a pneumatic cylinder and drawing an analogy between the conditions for maximum power in fluid and thermal power conversion. Based on this work, we extend the idea to an endoreversible compressor and, by analogy with the study of finite-time optimization of an endoreversible heat pump, we consider that a compressor is a system that provides mechanical work (or mechanical power) to a fluid to transfer it from a source with low energy potential to a sink with high energy potential (Figure 1). In our case, the flow is a gas flow rate, while the potential differences are pressure differences. Thus, the gas flow $\dot{V}$ is the product of a conductance *C* by a pressure difference ΔP. This notion of a tube as an impedance or resistance in the electrical analogy sense was introduced by Knudsen [52], and Dushman [53] defined the concept of conductance as the ratio between a fluid flow rate and a pressure difference. Conductance measures how easily flow occurs in response to a pressure differential: the higher the conductance for a given pressure differential, the greater the flow rate. In the classical endoreversible heat pump machine, the mechanical power ${\dot{W}}_{c}$ pumps the heat from the cold source at temperature $T_{C}$ to the hot sink at temperature $T_{H}$. The present endoreversible model may differ from the behavior of a real compressor due to irreversible mechanical losses in the moving parts of the compressor, rarefaction effects (Knudsen number > 0.001), pressure losses due to the surface condition, and roughness of the compressor wall materials. However, as a preliminary study, we will assume that these losses do not exist, focusing on an endoreversible model with irreversibilities only at the source and sink, and which are represented by the thermal conductances $C_{m}$ and $C_{M}$, respectively [34,35,36].

**Figure 2:**
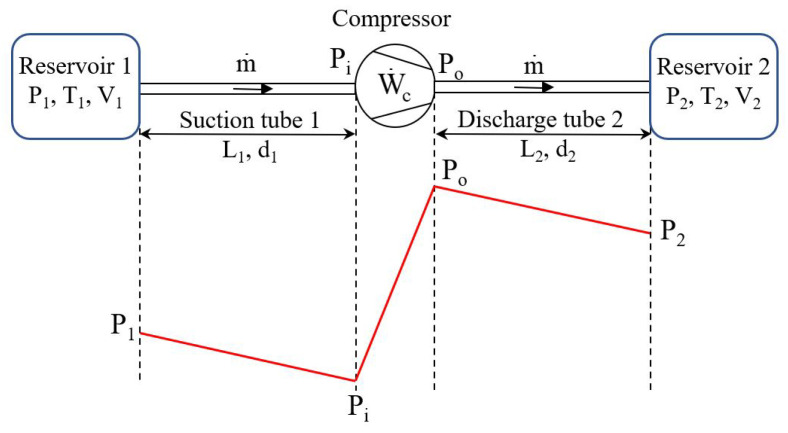
Schematics of a compressor. The compressor generates the mass flow $\dot{m}$ under the $\left(P_{o}-P_{i}\right)$ pressure difference. The pressure losses ($P_{i}-P_{1}$) and ($P_{2}-P_{o}$) are created by the fluid frictions in the suction and discharge tubes ($L_{1},d_{1}$) and ($L_{2},d_{2}$), respectively.

A compressor draws a gas through suction tube 1, of length $L_{1}$ and internal diameter $d_{1}$, from reservoir 1 (equivalent to the cold source of a heat pump) at the average pressure $P_{1}$, temperature $T_{1}$, and discharges it into reservoir 2 (equivalent to the hot sink of a heat pump) at the average pressure $P_{2}$ and temperature $T_{2}$ through discharge tube 2 of length $L_{2}$ and internal diameter $d_{2}$. The pressure at the compressor inlet is $P_{i}$ while the outlet pressure is $P_{o}$. The pressure losses ($P_{i}-P_{1}$) and ($P_{2}-P_{o}$) are created by the fluid frictions in the suction and discharge tubes, respectively. The fluid is supposed to be incompressible and fully developed, with a constant velocity profile throughout the tube length, and laminar. We impose a constant average mass flow rate of gas $\dot{m}$, without leaks, during the compression phase. We note ${\dot{W}}_{c}$, the mechanical compression power (Figure 2).

### 2.1. Optimal Mechanical Power of the Compressor

The Bernoulli equation written for suction tube 1 makes it possible to link the two pressures $P_{1}$ and $P_{i}$ by the following classical expression:

(1) $$P_{1}-P_{i}=\frac{8}{\pi^{2}}\frac{{\dot{m}}^{2}}{\rho_{1}\;d_{1}^{4}}\left[1+\lambda_{1}\frac{L_{1}}{d_{1}}\right].$$

With the mass flow inside pipe 1,

(2) $$\dot{m}=\rho_{1}\;S_{1}\;V_{1}.$$

Equations (1) and (2) are based on the assumption that the fluid can be treated as a continuum. However, for micro-sized tubes or under certain low-pressure conditions (where multiple characteristic length scales must be considered), the Knudsen number Kn is typically used to assess the validity of this assumption. Defined as the ratio between the mean free path *ℓ* (the average distance a molecule travels between collisions) and the characteristic length *L* (such as the hydraulic diameter of the tube), the Knudsen number helps determine the continuum nature of the flow. When Kn < 0.001, the continuum hypothesis holds, and Equations (1) and (2) remain applicable [54].

The tubes under consideration are characterized by small diameters ranging from 50 µm to 200 µm, and relatively long lengths between 50 mm and 100 mm. Although these dimensions suggest that rarefaction effects may be non-negligible—particularly given that the Knudsen number Kn exceeds 0.001—we will not introduce correction terms to account for velocity slip at the wall. This simplification is justified by the assumption that continuum flow remains a valid approximation within the operating conditions of the system. To limit friction losses in the suction and discharge tubes, a laminar flow condition is imposed; then, the pressure loss coefficient λ is expressed by the Blasius relation [55]:

(3) λ1=64Re1=16πμ1d1m˙.

Equation (3) into (1) gives

(4) $$P_{1}-P_{i}=\frac{8}{\pi^{2}}\frac{{\dot{m}}^{2}}{\rho_{1}\;d_{1}^{4}}\left[1+16\;\pi \;\frac{\mu_{1}\;L_{1}}{\dot{m}}\right].$$

Whatever the gas used (see Table 1), taking into account the condition relating to the viscous laminar flow regime, as well as the very low mass flow rates ($\dot{m}\leq$ $10^{-6}$ kg $s^{-1}$) and the dimensions of the suction and discharge tubes, we verify the relationship:

(5) $$16\;\pi \;\frac{\mu_{1}\;L_{1}}{\dot{m}}>>1.$$

The pressure difference $\left(P_{1}-P_{i}\right)$ is proportional to the volumetric flow rate $\dot{V_{1}}$ (Figure 1) or mass flow rate $\dot{m}=\;\rho_{1}\;\dot{V_{1}}$. In these conditions, Equation (4) becomes

(6) $$\Delta P_{1}=P_{1}-P_{i}=\frac{128}{\pi }\frac{\mu_{1}\;L_{1}}{\rho_{1}\;d_{1}^{4}}\dot{m}=\frac{128}{\pi }\frac{\mu_{1}\;L_{1}}{d_{1}^{4}}\dot{V_{1}}=\frac{\dot{V_{1}}}{C_{1}}$$

with

(7) $$\frac{1}{C_{1}}=\frac{128}{\pi }\frac{\mu_{1}\;L_{1}}{d_{1}^{4}}.$$

We define the factor $K_{1}=\frac{1}{\rho 1\;C_{1}}$ as an analog of a fluidic resistance, and the pressure difference $\Delta P_{1}$ becomes

(8) $$\Delta P_{1}=K_{1}\;\dot{m}$$

with

(9) $$K_{1}=\frac{128}{\pi }\frac{\mu_{1}\;L_{1}}{\rho_{1}\;d_{1}^{4}}.$$

Identical reasoning applied to the flow on the discharge side leads to the following relations:

(10) $$\Delta P_{2}=P_{o}-P_{2}=\frac{128}{\pi }\frac{\mu_{2}\;L_{2}}{\rho_{2}\;d_{2}^{4}}\dot{m}=\frac{128}{\pi }\frac{\mu_{2}\;L_{2}}{d_{2}^{4}}\dot{V_{2}}=\frac{\dot{V_{2}}}{C_{2}}$$

with

(11) $$\frac{1}{C_{2}}=\frac{128}{\pi }\frac{\mu_{2}\;L_{2}}{d_{2}^{4}}.$$

The factor $K_{2}=\frac{1}{\rho 2\;C_{2}}$ is defined as an analog of fluidic resistance, and the pressure difference $\Delta P_{2}$ becomes

(12) $$\Delta P_{2}=K_{2}\;\dot{m}$$

with

(13) $$K_{2}=\frac{128}{\pi }\frac{\mu_{2}\;L_{2}}{\rho_{2}\;d_{2}^{4}}.$$

The diameter of each tube significantly affects the calculation of pressure losses. In the laminar regime, the pressure loss ΔP varies linearly with the mass flow $\dot{m}$.

The mechanical power of a compressor being proportional to the variation in enthalpy of the gas, the power supplied by this compressor, noted ${\dot{W}}_{c}$, related to the mass flow $q_{m}$, is written as follows:

(14) $${\dot{W}}_{c}=\frac{\dot{m}\;c_{p}\;T_{1}}{\eta_{c}}\left[{\left(\frac{P_{o}}{P_{i}}\right)}^{\frac{\gamma -1}{\gamma }}-1\right],$$

where $\eta_{c}$ is the isentropic compression efficiency. The endoreversible compressor assumes adiabatic efficiency $\eta_{c}$ = 1, to maintain the analogy with an endoreversible heat pump. It means there are no energy losses to compress the gas from the pressure $P_{i}$ to $P_{o}$ inside the compressor. Otherwise, we would have an irreversible compressor. Equations (6) and (10) are reported into Equation (14), and

(15) $${\dot{W}}_{c}=\dot{m}\;c_{p}\;T_{1}\left[{\left(\frac{P_{2}+\Delta P_{2}}{P_{1}-\Delta P_{1}}\right)}^{\frac{\gamma -1}{\gamma }}-1\right].$$

Let us introduce the pressure ratio τ by

(16) $$\tau ={\left(\frac{P_{2}+\Delta P_{2}}{P_{1}-\Delta P_{1}}\right)}^{\frac{\gamma -1}{\gamma }}.$$

The expression (16) is simplified if we assume that the pressure losses are negligible compared to the average pressure values and $\Delta P_{1}<P_{1}$ and $\Delta P_{2}<P_{2}$. By an expansion limited to the first order of the numerator and the denominator of the relation (16), we finally obtain

(17) $$\tau \approx \left(1+\frac{\gamma -1}{\gamma }\frac{\Delta P_{1}}{P_{1}}\right)\left(1+\frac{\gamma -1}{\gamma }\frac{\Delta P_{2}}{P_{2}}\right){\left(\frac{P_{2}}{P_{1}}\right)}^{\frac{\gamma -1}{\gamma }}$$

and, neglecting the second order terms $\left(\frac{\Delta P_{1}}{\Delta P_{1}}\frac{\Delta P_{2}}{\Delta P_{2}}\right)$, the pressure ratio is

(18) $$\tau \approx 1+\frac{\gamma -1}{\gamma }\frac{\Delta P_{1}}{P_{1}}\left(1+\frac{P_{1}}{P_{2}}\frac{\Delta P_{2}}{\Delta P_{1}}\right).$$

The expression of the compressor power ${\dot{W}}_{c}$ is written as follows:

(19) $${\dot{W}}_{c}=\dot{m}\;c_{p}\;T_{1}\{\left[1+\frac{\gamma -1}{\gamma }\frac{\Delta P_{1}}{P_{1}}\left(1+\frac{P_{1}}{P_{2}}\frac{\Delta P_{2}}{\Delta P_{1}}\right)\right]{\left(\frac{P_{2}}{P_{1}}\right)}^{\frac{\gamma -1}{\gamma }}-1\},$$

and

(20) $$\frac{\Delta P_{2}}{\Delta P_{1}}=\frac{\mu_{2}\;L_{2}\;T_{2}}{\mu_{1}\;L_{1}\;T_{1}}{\left(\frac{d_{1}}{d_{2}}\right)}^{4}{\left(\frac{P_{1}}{P_{2}}\right)}^{2}.$$

The volumes of the two reservoirs are sufficiently large compared to those of the tubes to consider that the temperatures $T_{1}$ and $T_{2}$ are equal but that only the pressures $P_{1}$ and $P_{2}$ are different. And if $T_{1}=T_{2}$ then $\mu_{1}=\mu_{2}$ and $\rho_{1}=\rho_{2}$, and the pressure ratio $\frac{\Delta P_{2}}{\Delta P_{1}}$ yields

(21) $$\frac{\Delta P_{2}}{\Delta P_{1}}\approx \frac{L_{2}}{L_{1}}{\left(\frac{d_{1}}{d_{2}}\right)}^{4}{\left(\frac{P_{1}}{P_{2}}\right)}^{2}\approx \frac{K_{2}}{K_{1}}\;{\left(\frac{P_{1}}{P_{2}}\right)}^{2},$$

where the ratio $K_{2}/K_{1}$ is a function of geometric parameters (lengths and diameters) only:

(22) $$\frac{K_{2}}{K_{1}}=\frac{L_{2}}{L_{1}}{\left(\frac{d_{1}}{d_{2}}\right)}^{4}.$$

Finally, the compressor power ${\dot{W}}_{c}$ is written as follows:

(23) $${\dot{W}}_{c}\approx \dot{m}\;c_{p}\;T_{1}\{\left[1+\frac{\gamma -1}{\gamma }\;\left(1+\frac{P_{1}}{P_{2}}\;\frac{K_{2}}{K_{1}}\right)\;\frac{K_{1}\;\dot{m}}{P_{1}}\right]\;{\left(\frac{P_{2}}{P_{1}}\right)}^{\frac{\gamma -1}{\gamma }}-1\}.$$

(24) $${\dot{W}}_{c}\approx \dot{m}\;c_{p}\;T_{1}\{\left[1+\frac{128}{\pi }\frac{\gamma -1}{\gamma }\frac{\mu_{1}\;r\;T_{1}}{P_{1}^{2}}\frac{L_{1}}{d_{1}^{4}}\left(1+\frac{L_{2}}{L_{1}}{\left(\frac{d_{1}}{d_{2}}\right)}^{4}{\left(\frac{P_{1}}{P_{2}}\right)}^{3}\right)\dot{m}\right]{\left(\frac{P_{2}}{P_{1}}\right)}^{\frac{\gamma -1}{\gamma }}-1\}.$$

We define the normalized compression power ${\dot{W}}_{c}^{*}$ as a normalized power from Equation (24):

(25) $${\dot{W}}_{c}^{*}=\frac{{\dot{W}}_{c}}{\dot{m}\;c_{p}\;T_{1}}.$$

**Figure 3:**
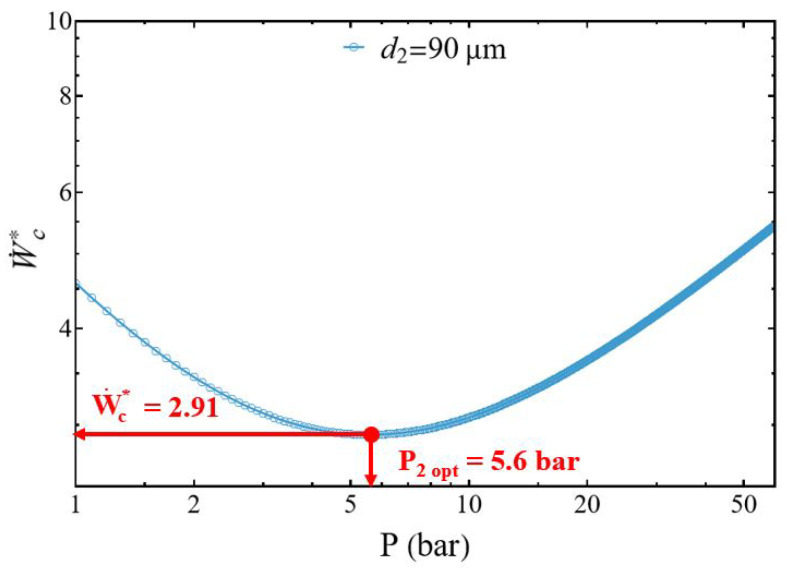
Evolution of the normalized compressor power as a function of the discharge pressure for $d_{1}/d_{2}$ > 1. Conditions: helium (Table 1), discharge tube diameter $d_{2}$ = 90 μm, $d_{1}$ = 200 μm, $L_{1}=L_{2}=0.05$ m.

The normalized power ${\dot{W}}_{c}^{*}$ presents a minimal value for an optimal reservoir pressure $P_{2opt}$ that must be determined (Figure 3). To do this, let us cancel the first derivative of Equation (23), $\frac{\partial {\dot{W}}_{c}^{*}}{\partial P_{2}}=0$, and it becomes

(26) $$P_{2opt}=\frac{K_{2}\;\dot{m}}{\gamma \;P_{1}+\left(\gamma -1\right)\;K_{1}\;\dot{m}}\;P_{1}.$$

The optimal normalized power is finally written:

(27) $${\left.{\dot{W}}_{c}^{*}\right|}_{opt}=\frac{{\left.{\dot{W}}_{c}\right|}_{opt}}{\dot{m}\;c_{p}\;T_{1}}.$$

(28) $${\left.{\dot{W}}_{c}^{*}\right|}_{opt}=\;{\left[\frac{\gamma -1}{\gamma }\;\frac{1}{P_{1}^{2}}{\left(\frac{P_{2}}{P_{1}}\right)}^{-\frac{1}{\gamma }}\right]}^{\frac{\gamma }{\gamma -1}}\;\left[1+\frac{\gamma -1}{\gamma }\;\frac{K_{1}\dot{m}}{P_{1}}+K_{2}\;\dot{m}P_{1}{\left(\frac{P_{2}}{P_{1}}\right)}^{\frac{1}{\gamma }}\right]-1.$$

Indeed, we show that the second derivative of the power $\frac{\partial^{2}{\dot{W}}_{c}^{*}}{\partial {P_{2}}^{2}}$ is positive, confirming a minimal point corresponding to $P_{2opt}$, and it has the following expression:

(29) $$\frac{\partial^{2}{\dot{W}}_{c}^{*}}{\partial {P_{2}}^{2}}=\frac{1}{{\left(P_{1}P_{2}\gamma \right)}^{2}}\left\{{\left(\frac{P_{2}}{P_{1}}\right)}^{-1/\gamma }\frac{\gamma -1}{\gamma }\left[K_{2}P_{1}\dot{m}\left(\gamma +1\right)\right]-P_{2}\left[K_{1}\dot{m}\frac{\gamma -1}{\gamma }+P_{1}\right]\right\}.$$

The compressor’s power curve evolves in two different ways. In the first phase, the power decreases with the discharge pressure $P_{2}$ up to a minimum corresponding to $P_{2opt}$, for which $\frac{\partial^{2}{\dot{W}}_{c}^{*}}{\partial {P_{2}}^{2}}$ is positive. When $d_{1}\geq \;d_{2}\;=1$, to maintain the mass flow $\dot{m}$ constant, the power of the compressor decreases and reaches a minimum point. Then, from this point, the power increases with pressure $P_{2}$ due to the logical increase in pressure losses in the discharge tube.

### 2.2. Energy Conversion Efficiency of the Compressor

By analogy with the coefficient of performance of a heat pump, the energy conversion efficiency ϵ of the compressor is written as the ratio between the power required to compress the gas from $P_{o}$ to $P_{2}$ corresponding to the viscous power ${\dot{W}}_{visc\;2}$ expended along the discharge tube, and the total power ${\dot{W}}_{c}$ supplied to the compressor to compress the gas from $P_{1}$ to $P_{2}$ (Figure 1):

(30) $$\varepsilon =\frac{{\dot{W}}_{visc\;2}}{{\dot{W}}_{c}}.$$

Considering the perfect gas law and Equation (12), the viscous power is

(31) $${\dot{W}}_{visc\;2}=\dot{V_{2}}\;\Delta P_{2}=\frac{K_{2}}{\rho_{2}}\;{\dot{m}}^{2}=c_{p}\frac{\gamma -1}{\gamma }\;K_{2}\;\frac{T_{2}}{P_{2}}\;{\dot{m}}^{2}.$$

From relations (24) and (31), for a given type of gas (adiabatic index γ) and its mass flow rate $\dot{m}$ the energy conversion efficiency ϵ of the compressor (30) is written as a function of the main dimensional parameters, the pressures of the reservoirs, $P_{1}$ and $P_{2}$, and the conductances of the tubes, $K_{1}$ and $K_{2}$:

(32) $$\varepsilon =\frac{K_{2}\;\frac{\gamma -1}{\gamma }\;\dot{m}}{P_{2}\left\{\;\left[1+\frac{\gamma -1}{\gamma }\;\left(1+\frac{P_{1}}{P_{2}}\;\frac{K_{2}}{K_{1}}\right)\;\frac{K_{1}\;\dot{m}}{P_{1}}\right]\;{\left(\frac{P_{2}}{P_{1}}\right)}^{\frac{\gamma -1}{\gamma }}-1\right\}}.$$

## 3. Results and Discussion

In this section, we address the parametric study of compressor performance for different pressure levels and different tube diameters. We will assume that the gas mass flow rate remains constant ($\dot{m}=0.3\;\mu$g $s^{-1}$) and that the tube lengths also remain constant ($L_{1}=L_{2}=50$ mm). All simulations were carried out with helium, and the influence of the nature of the gases (Table 1) on the performance of the endoreversible compressor will only be considered in Section 3.2.3.

### 3.1. Compressor Power

**Figure 4:**
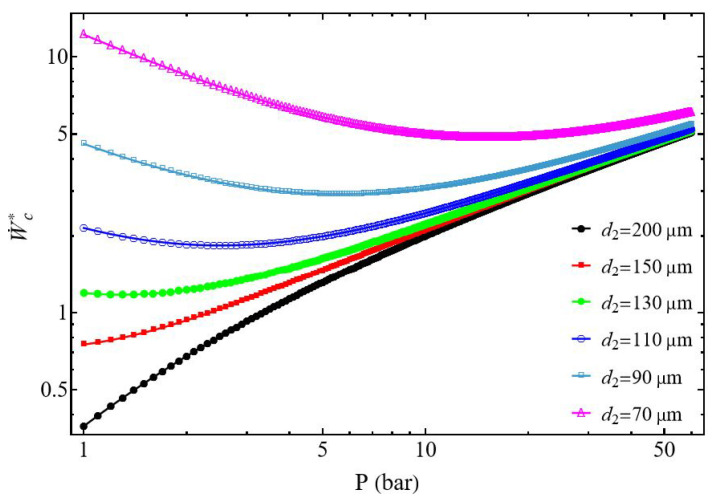
Evolution of the normalized compressor power as a function of the discharge pressure. Influence of the discharge tube diameter $d_{2}$ for $d_{1}/d_{2}\;\geq \;1$. Conditions: helium (Table 1), $d_{1}$ = 200 µm, $L_{1}=L_{2}$ = 50 mm, and $\dot{m}$ = 0.3 µg $s^{-1}$.

The study of the evolution of the compressor power ${\dot{W}}_{c}^{*}$ as a function of the discharge pressure presents two different behaviors. The normalized power ${\dot{W}}_{c}^{*}$ increases with the pressure $P_{2}$, but also when the diameter $d_{2}$ of the discharge tube decreases (Figure 4). However, for each diameter $d_{2}$ there is a specific pressure at which compression power reaches its minimum.

**Figure 5:**
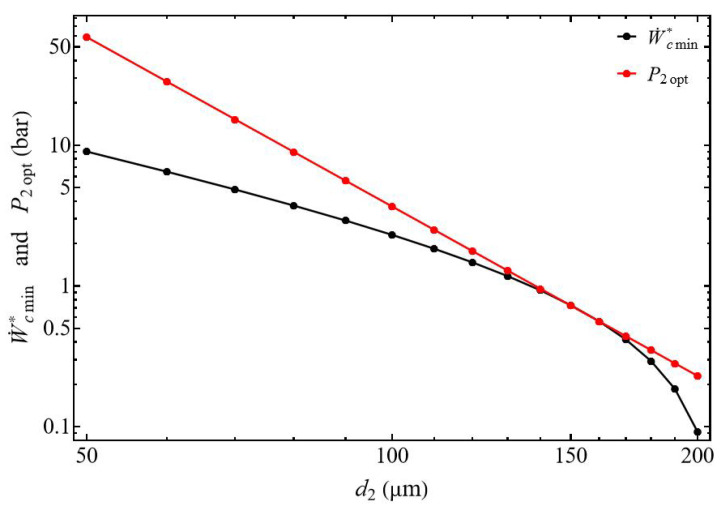
Evolution of normalized power and optimal compressor outlet pressure. Influence of the discharge tube diameter $d_{2}$ for $d_{1}/d2\;\geq \;1$. Conditions: helium (Table 1), $L_{1}=L_{2}$ = 50 mm, and $\dot{m}$ = 0.3 µg $s^{-1}$.

When the diameter ratio $d_{1}/d_{2}\;\geq \;1$ the power of the compressor presents a minimum for each discharge tube diameter $d_{2}$ (Figure 4). This minimum slides with the pressure and increases when the tube diameter $d_{2}$ decreases, corresponding to the impact of the pressure losses (Figure 5). The normalized power ${\dot{W}}_{c\;min}^{*}$ decreases when the diameter $d_{2}$ of the discharge tube increases logically because for the same mass flow $\dot{m}$ the fluid velocity of the gas decreases, generating a corresponding decrease of the pressure losses.

We show $\frac{\partial^{2}{\dot{W}}_{c}^{*}}{\partial {P_{2}}^{2}}>0$, and the normalized power ${\dot{W}}_{c}^{*}$ presents a minimal value ${\dot{W}}_{c\;min}^{*}$ for the pressure $P_{2opt}$ (Equation (26)), and this for all delivery diameter values $d_{2}$ from 70 to 200 µm (with $d_{1}$ = 200 µm), and this for all discharge diameter values. Under these conditions, for a given tank pressure $P_{2}$ and a given mass flow rate $\dot{m}$ we can choose the suction tube diameter allowing the gas to be compressed with a minimum of energy. Figure 5 shows the monotonic decrease of the optimal pressure $P_{2opt}$ as a function of the diameter $d_{2}$. It can be observed that the optimal power collapses, starting from a diameter of $d_{2}$ = 150 µm.

**Figure 6:**
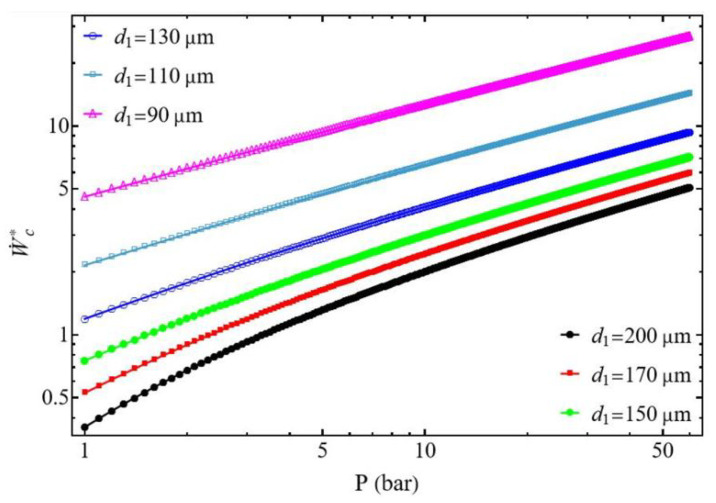
Evolution of the normalized compressor power as a function of the discharge pressure. Influence of the suction tube diameter $d_{1}$ for $d_{1}/d_{2}\;\leq \;1$. Conditions: helium Table 1), $d_{2}$ = 200 µm, $L_{1}=L_{2}$ = 50 mm, and $\dot{m}$ = 0.3 µg $s^{-1}$.

**Figure 7:**
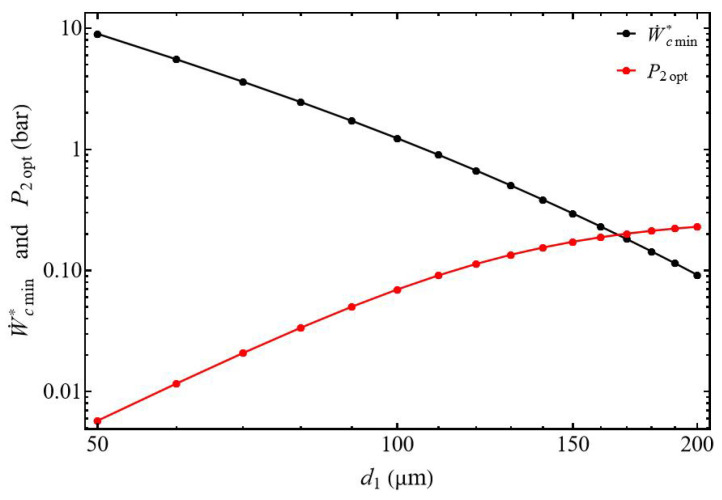
Evolution of normalized power and optimal compressor outlet pressure. Influence of the suction tube diameter $d_{1}$ for $d_{1}/d_{2}\;\leq \;1$. Conditions: helium (Table 1),$L_{1}=L_{2}$ = 50 mm, and $\dot{m}$ = 0.3 µg $s^{-1}$.

When the diameter ratio $d_{1}/d_{2}\;\leq \;1$, the power of the compressor ${\dot{W}}_{c}^{*}$ does not present minimum values but increases continuously with the pressure and also when the diameter $d_{1}$ of the suction tube decreases due to the increase in pressure drops (Figure 6). The compressor’s power does not reveal an optimal value that can be used for sizing the compressor. The normalized power ${\dot{W}}_{c\;min}^{*}$ (Figure 7) decreases when the diameter $d_{1}$ of the suction tube increases logically because for the same value of the mass flow $\dot{m}$ the tube section presents a bigger area and then a lower velocity. In this configuration, the optimal pressure $P_{2opt}$ increases with the rise in diameter $d_{1}$ and tends toward a value of $P_{2optlim}$ = 0.22 bar. This demonstrates a behavior very different from the case where $d_{1}/d_{2}\;\geq \;1$ (Figure 5).

### 3.2. Compressor Efficiency

The compressor efficiency represents the ability of the compressor to convert the power input ${\dot{W}}_{c}^{*}$ to the power ${\dot{W}}_{visc\;2}$ to compress the fluid from the pressure $P_{1}$ to $P_{2}$. The influences of the suction diameter tube $d_{1}$ and the discharge diameter tube $d_{2}$ are analyzed as a function of the pressure. First, we will analyze the compression efficiency as a function of discharge pressure and then as a function of compression power.

#### 3.2.1. Effect of the Discharged Pressure $P_{2}$

**Figure 8:**
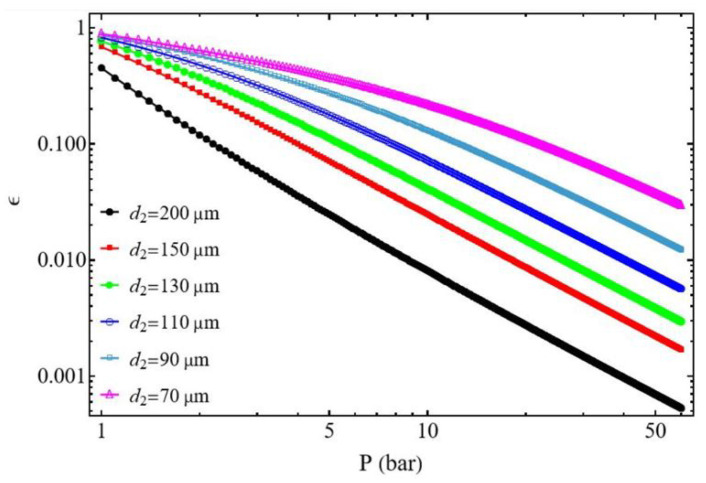
Compressor efficiency. Influence of the discharge tube diameter $d_{2}$. Conditions: helium (Table 1), $d_{1}$ = 200 µm for $d_{1}/d_{2}$ ≥ 1, $L_{1}=L_{2}$ = 50 mm, and $\dot{m}$ = 0.3 µg $s^{-1}$.

**Figure 9:**
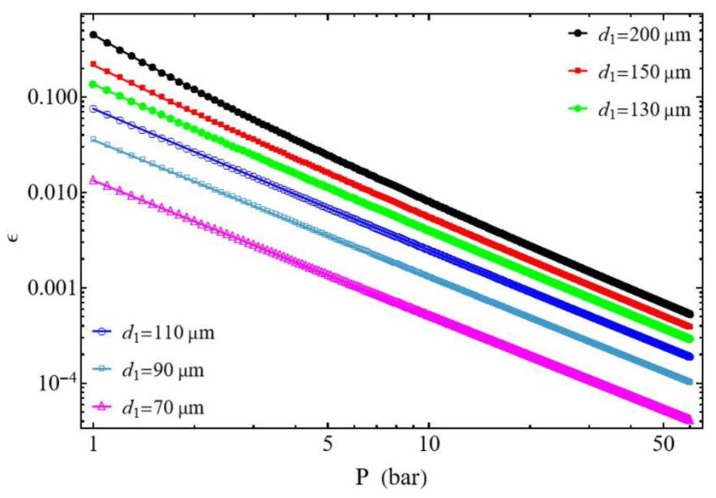
Compressor efficiency. Influence of the suction tube diameter $d_{1}$. Conditions: helium (Table 1), $d_{2}$ = 200 µm for $d_{1}/d_{2}$ ≤ 1, $L_{1}=L_{2}$ = 50 mm, and $\dot{m}$ = 0.3 µg $s^{-1}$.

In Figure 8 and Figure 9, the efficiency ε decreases with pressure. Overall, for the same mass flow rate $\dot{m}$ this can be explained by the increase in pressure losses as the diameter of the suction and discharge tubes decreases. However, the compressor behaves very differently, depending on the ratio $d_{1}/d_{2}$. The compression efficiency for the same pressure value, $P_{2}$, is higher for the case $d_{1}/d_{2}\;\geq \;1$ than for the case $d_{1}/d_{2}\;\leq \;1$. For the case $d_{1}/d_{2}\;\geq \;1$, it is shown that at the same pressure, $P_{2}$, the efficiency decreases as the diameter $d_{2}$ of the discharge tube increases. Conversely, for the case $d_{1}/d_{2}\;\leq \;1$ the efficiency decreases as the diameter $d_{1}$ of the suction tube decreases. This phenomenon can be attributed to the compressor’s constant mass flow rate $\dot{m}$ during the compression process. In general, for the case $d_{1}/d_{2}\;\geq \;1$ the compressor demonstrates the highest efficiency values.

#### 3.2.2. Bounds of Efficiency and Compression Power

**Figure 10:**
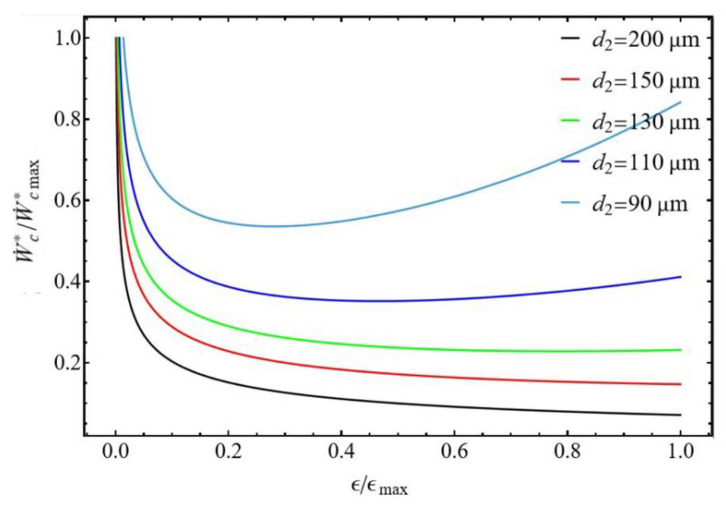
Normalized power versus normalized compressor efficiency. Influence of the discharge tube diameter $d_{2}$. Conditions: helium (Table 1), $d_{1}$ = 200 µm for $d_{1}/d_{2}$ ≤ 1, $L_{1}=L_{2}$ = 50 mm, and $\dot{m}$ = 0.3 µg $s^{-1}$.

**Figure 11:**
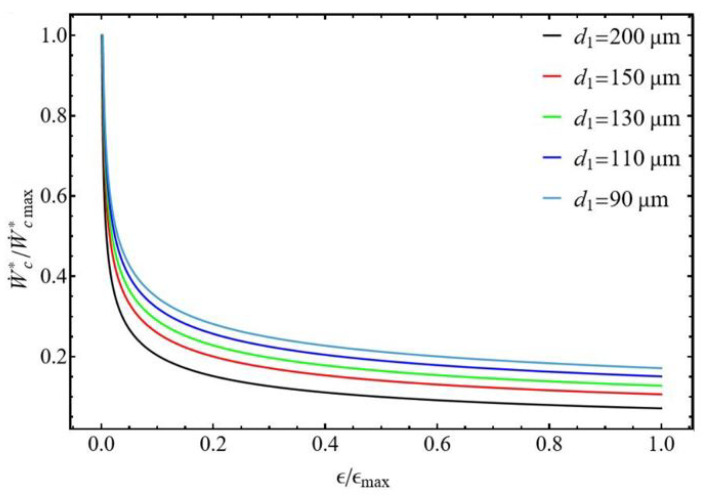
Normalized power versus normalized compressor efficiency. Influence of the suction tube diameter $d_{1}$. Conditions: helium (Table 1), $d_{2}$ = 200 µm for $d_{1}/d_{2}$ ≥ 1, $L_{1}=L_{2}$ = 50 mm, and $\dot{m}$ = 0.3 µg $s^{-1}$.

The compressor’s performance can be characterized by analyzing the normalized compression power as a function of normalized efficiency. The effects of the ratios $d_{1}/d_{2}$ were assessed, and the results are displayed in Figure 10 and Figure 11. Both figures exhibit the same remarkable point. For the ratio $\epsilon /\epsilon_{max}$ close to zero, the compressor delivers maximum power to compensate for its very low efficiency to maintain the desired mass flow rate $\dot{m}$ and compression ratio $P_{2}/P_{1}$. On the other hand, at a given normalized efficiency the normalized power of the compressor increases when the diameter of the tubes decreases. This is explained by the fact that the compressor must compensate for the pressure losses, which increase at constant mass flow rate when the tube diameters decrease.

For the diameter ratio $d_{1}/d_{2}\;\leq \;1$ (Figure 10), the normalized power shows a minimum for some tube diameter values $d_{2}\;\leq \;110\;\mu$m corresponding to a range of normalized efficiency $\epsilon /\epsilon_{max}$ between 0.2 and 0.4. For diameters $d_{2}>110\;\mu$m, the normalized power of the compressor decreases with normalized efficiency. For the diameter ratio $d_{1}/d_{2}\;\geq \;1$ (Figure 11), the normalized power constantly decreases with normalized efficiency but does not present a minimum.

#### 3.2.3. Influence of Gas Type

**Figure 12:**
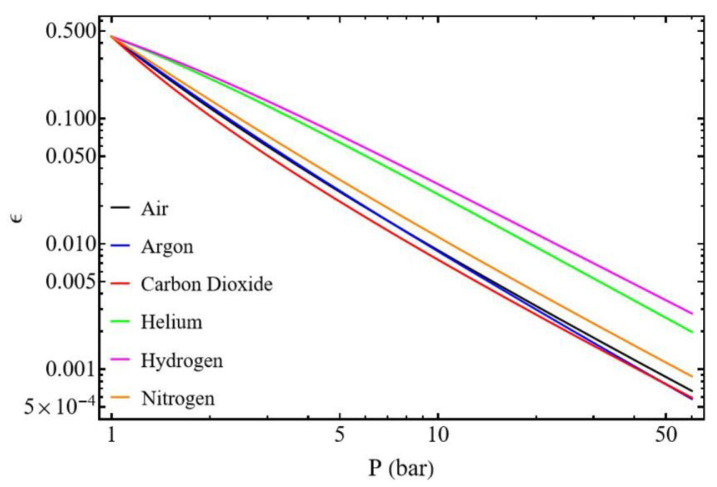
Compressor efficiency. Influence of the thermophysical properties of the gas (Table 1) with $d_{1}$ = 200 µm, $d_{2}$ = 200 µm, $L_{1}$ = 50 mm, $L_{2}$ = 50 mm, and $\dot{m}$ = 0.3 µg $s^{-1}$.

The nature of the gas significantly influences compressor performance (Table 1). Figure 12 illustrates two major points. The efficiency decreases with the increase of the pressure for all gases, and hydrogen and helium yield the highest compressor efficiency. These gases are lightweight, possess high thermal capacities, and have exceptionally low molar masses, which facilitates their flow through the tubing. At 1 bar, gas type has minimal impact on compressor efficiency (ϵ = 0.45). However, as pressure increases, reaching 5 bar for instance, compressor efficiency for hydrogen and helium is already twice that of other gases.

## 4. Conclusions

We have used finite-time thermodynamics to carry out a parametric study of the performance of an endoreversible compressor by analogy with an endoreversible heat pump. This model cleanly separates internal reversible processes from external irreversibilities (like finite heat transfer rates for the heat pump, finite pressure losses for the compressor). This lets us pinpoint sources of performance loss (power, efficiency) more effectively due to the diameter ratios, tube lengths, mass flows, pressures, and type of gases. This model yields analytical solutions that are easier to interpret and optimize in preliminary design. The compressor compresses a gas reversibly at steady-state conditions, and external irreversibilities occur between the compressor and the low- and high-pressure reservoirs, along the suction and discharge tubes, respectively. Compressor performance is addressed through the evolution of normalized power and normalized compression efficiency. The compressor operates at a constant mass flow rate in the laminar regime. We have shown that the power consumed by the compressor increases with the discharge pressure, due to the increase in pressure losses in the suction and discharge tubes, and that the compression efficiency logically decreases as this pressure increases. The influence of the conductances $K_{1}$ and $K_{2}$, considered in terms of the diameters of the suction tube $d_{1}$ and discharge tube $d_{2}$, is important, particularly through the ratio $d_{1}/d_{2}$, which can be greater or less than unity. Although the compressor consumes less power when $d_{1}/d_{2}\leq 1$, the compression efficiency improves when $d_{1}/d_{2}\geq 1$, provided that $d_{1}\leq 110\;\mu$m and $d_{2}=200\;\mu$m. Finally, the analysis showed that performance (power and efficiency) is best for low-molar mass gases such as helium and hydrogen. Finite-time thermodynamics is a powerful tool for analyzing the behavior and sizing of an energy conversion system, such as a compressor, and defining its performance limits. This study holds significant technological relevance, as miniaturizing mechanical and pneumatic components presents a major industrial and scientific challenge, particularly within the domain of micro-electromechanical systems (MEMSs).

## Figures and Tables

**Table 1:** Thermophysical properties of different gases at 20 °C and standard atmospheric pressure 101,325 Pa [56,57,58,59].

Gas	Dynamic Viscosity μ ($10^{-5}$ Pa s)	Density ρ (${\mathrm{kgm}}^{-3}$)	Specific Heat $c_{p}$ (J ${\mathrm{kg}}^{-1}$ $K^{-1}$)	Thermal Conductivity λ (W $m^{-1}$ $K^{-1}$)	Adiabatic Index γ	Molar Mass M (kg ${\mathrm{kmol}}^{-1}$)
Air	1.817	1.203	1015	0.02565	1.400	28.97
Argon (Ar)	2.240	1.661	520	0.01737	1.670	39.948
Carbon dioxide (${\mathrm{CO}}_{2}$)	1.493	1.871	851	0.01626	1.294	44.009
Helium (He)	1.973	0.166	5196	0.14929	1.666	4.003
Hydrogen ($H_{2}$)	0.867	0.084	14285	0.17690	1.410	2.016
Nitrogen ($N_{2}$)	1.757	1.164	1040	0.02543	1.400	28.013

## Data Availability

Data is contained within the article.
